# An examination into the effects of tocotrienols (TheraPrimE® rice) on cognitive abilities and sleep in healthy adults: a randomised, double-blind, placebo-controlled trial

**DOI:** 10.3389/fnut.2025.1621516

**Published:** 2025-09-03

**Authors:** Adrian L. Lopresti, Stephen J. Smith, Lixin Ding, Yanmei Li, Peinan Zhang

**Affiliations:** ^1^Clinical Research Australia, Perth, WA, Australia; ^2^College of Science, Health, Engineering and Education, Murdoch University, Perth, WA, Australia; ^3^BGG Americas, Irvine, CA, United States; ^4^BGG, Beijing, China

**Keywords:** tocotrienols, vitamin E, memory, cognition, sleep

## Abstract

**Background/objectives:**

Tocotrienols are a form of vitamin E that may have neuroprotective effects. However, there have been no studies examining its effects on cognitive function when delivered as a stand-alone intervention. The purpose of this two-arm, 12-week, randomised, double-blind, placebo-controlled trial was to examine the effects of supplementation with tocotrienols derived from rice bran (TheraPrimE® rice) on memory and sleep in adults with subjective memory complaints.

**Methods:**

Ninety-one adults aged 40–80 were supplemented with a placebo or 100 mg of tocotrienols daily. Outcome measures included the Test of Memory and Learning (version 2), and self-report questionnaires assessing executive function and sleep quality. Moreover, changes in blood markers associated with inflammation, oxidative stress, and neurotropic activity were examined.

**Results:**

Compared to the placebo, tocotrienol supplementation was associated with greater improvements in general memory (*p* = 0.045, 95% CI: 0.34, 32.21). Memory changes were primarily due to improvements in non-verbal memory (*p* = 0.039, 95% CI: 0.68, 26.63). However, there were no group differences in changes in verbal memory. Moreover, there were no group differences in changes in self-reported executive function, although there were greater improvements in sleep disturbance in the tocotrienols group (*p* = 0.015, 95% CI: −4.80, −0.55). An examination of blood markers revealed a statistically significant larger increase in Tumour Necrosis Factor-α in the placebo group (*p* = 0.043) and a larger increase in C-reactive protein (*p* = 0.039) in the tocotrienols group. Tocotrienols were not associated with any serious adverse reactions.

**Conclusion:**

This is the first controlled study demonstrating the cognitive-enhancing and sleep-promoting effects of stand-alone supplementation with tocotrienols. However, future research is required to substantiate this study’s results and examine the potential mechanisms of action.

**Clinical trial registration:**

https://www.anzctr.org.au/ACTRN12624000351516.aspx, Identifier ACTRN12624000351516.

## 1 Introduction

In the general population, subjective memory complaints (SMC) are associated with a reduced global quality of life, increased risk of depression, and negative impact on daily living activities (1, 2). Older people with SMC are twice as likely to develop dementia compared to individuals without SMC. Approximately 2.3 and 6.6% of older people with SMC will progress to dementia and mild cognitive impairment (MCI), respectively every year (3). Research also suggests that SMC in middle age is a concern as it is associated with cortical thinning in brain regions affected by Alzheimer’s disease (AD) and poorer performance on objective memory tests (4). Memory complaints are distressing for adults of all ages and are associated with greater negative affect (5, 6). Therefore, identifying interventions to reduce the prevalence of SMC and the potential progression into worsening memory-related conditions is prudent.

Diet has been shown to protect against MCI and dementia, with antioxidant-rich foods having specific merit (7, 8). Vitamin E is an antioxidant nutrient that has received some attention for its cognitive-enhancing and neuroprotective effects. Vitamin E is classified into two major groups known as tocotrienols and tocopherols. Each group is further divided into alpha (α), beta (β), gamma (γ), and delta (δ) homologues based on the position of the methyl side changes on the chromanol ring. Tocopherols and tocotrienols differ in their side chain structures, where tocopherols have a saturated phytyl side chain attached to their chromanol ring, and tocotrienols possess an unsaturated isoprenoid side chain (9). High concentrations of tocopherols are found in lipid-rich plant products and common vegetable oils, while tocotrienols are found abundantly in some cereals, rice bran oil, and palm oil (10).

Research into the relationship between SMC and vitamin E is limited, although there is evidence from cross-sectional and longitudinal studies that a higher vitamin E intake and higher blood concentrations of vitamin E are associated with better cognitive function and a reduced prevalence of AD and cognitive decline. In a meta-analysis of 31 studies, it was concluded that compared to healthy controls, individuals with AD or age-related cognitive decline had lower circulatory concentrations of α-tocopherol (11). However, findings from randomised controlled studies in clinical populations have been mixed. In a review of four trials comprising adults with AD and MCI, it was concluded that there was no evidence that vitamin E in the form of α-tocopherol given to people with MCI prevented progression to dementia or improved cognitive function in people with MCI or AD. However, there was moderate-quality evidence from a single study that it may slow functional decline in AD (12). This inconsistent evidence may result from conducting trials on adults with established dementia, where the benefits from supplementation may be limited. Moreover, most trials have been undertaken using tocopherols, particularly α-tocopherols. In recent years, there has been an increased interest in tocotrienols as research suggests they may have higher physiological activity, including possessing greater neuroprotective effects and antioxidant potential than tocopherols (9, 13–16). As tocotrienols possess an unsaturated isoprenoid side chain, their mobility in cell membranes is enhanced, which potentially allows greater distribution in brain, skin, and liver tissue. Tocotrienol availability in selective brain regions has been associated with structural protection, particularly in white matter (17). Randomised controlled trials on the cognitive-enhancing effects of tocotrienols have demonstrated some positive effects in children with attention-deficit hyperactivity disorder (ADHD) (18), and on composite and verbal memory in healthy adults when administered in conjunction with astaxanthin (19). While there is some preliminary positive evidence of vitamin E supplementation in different populations, there have been no trials examining its effects as a stand-alone agent on cognitive function in adults with SMC.

Therefore, the primary objective of this study was to examine the effects of supplementation with tocotrienols (derived from rice bran) on cognitive performance in healthy adults with SMC. A secondary objective was to examine its effects on sleep quality. The relationship between vitamin E and sleep is insufficiently explored and given the important role sleep has on cognitive function (20) it is worthy of exploration. Increased oxidative stress and inflammation are associated with poorer sleep quality (21, 22), and potentially through these mechanisms, tocotrienols may have sleep-promoting effects. Finally, another secondary objective was to investigate potential mechanisms of action associated with tocotrienol supplementation by examining changes in blood markers associated with oxidative stress, inflammation, and neurotrophic activity. It was hypothesised that tocotrienol supplementation in adults with SMC would be associated with improvements in memory and sleep quality, and this may be via its antioxidant, anti-inflammatory, or neurotrophic-promoting activity.

## 2 Materials and methods

### 2.1 Study design and procedures

Ethics approval was received from the National Institute of Integrative Medicine Human Research Ethics Committee (approval number 0136E_2024), and informed consent was acquired from all participants. This trial was registered prospectively with the Australian and New Zealand Clinical Trials Registry (ACTRN12624000351516).

The recruitment of volunteers occurred between June and September 2024, through social media and emails to a database of interested volunteers. Participants completed an online screening questionnaire, where they provided demographic information, details of their health status, and medication intake. Volunteers also completed the Patient Health Questionnaire 4, which is a 4-item, validated self-report screening tool for depression and anxiety in adults (23). Respondents scoring 8 or more were ineligible for a phone interview as this indicated severe symptoms of depression and/or anxiety.

If deemed potentially suitable, volunteers were contacted by a researcher for a telephone interview. During this interview, a more comprehensive assessment of the eligibility criteria was undertaken, relevant demographic and sociographic details were obtained, and a full explanation of the study was provided. Moreover, the Modified Telephone Interview for Cognitive Status (TICS-M) was also administered. The TICS-M is a brief validated, interviewer-administered screening tool for cognitive impairment (24). If eligibility criteria were fulfilled, participants were emailed a copy of the consent form which needed to be completed electronically before commencement of the study. Participants attended visit 1 approximately 3–14 days after the telephone interview, where all assessments were conducted between 8 and 11 am after an overnight food fast. During visit 1, participants completed the cognitive assessment (Test of Memory and Learning-2), self-report questionnaires, and had a fasting blood sample collected for measurement of relevant plasma biomarkers.

### 2.2 Randomisation and blinding

**Figure 1:**
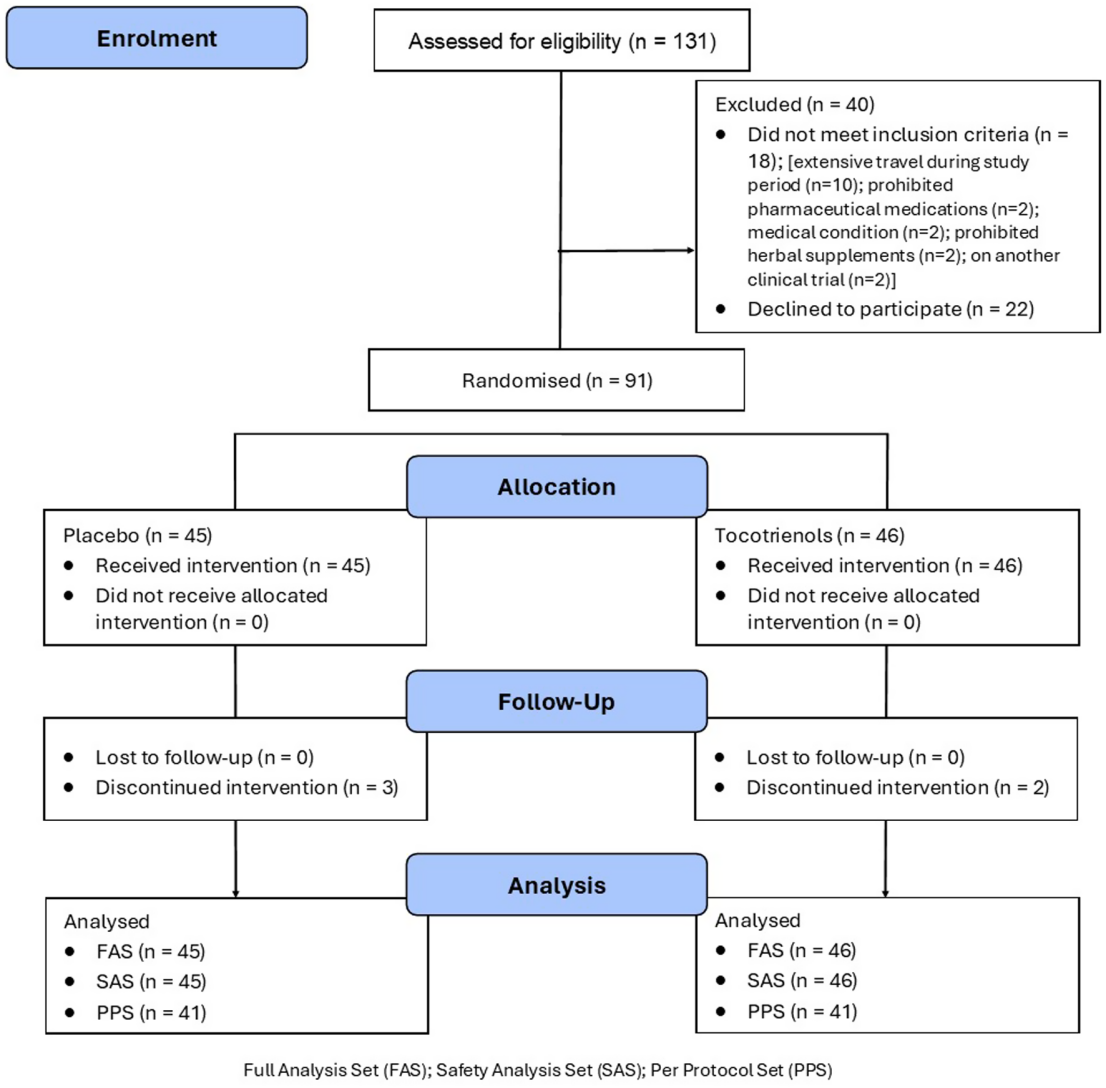
Systematic illustration of study design.

This was a 12-week, two-arm, parallel-group, randomised, double-blind, placebo-controlled trial (Figure 1). Participants were blinded during the study, and researchers and the statistician were blind to the treatment allocation until all outcomes were collected and a blind review was completed. The statistician remained blind to treatment allocation until the final analysis was completed. Participants were randomly allocated to one of two groups (tocotrienols or placebo) on a 1:1 ratio using a randomisation calculator with the randomisation structure comprising 9 permuted blocks, with 10 participants per block. A participant number was allocated based on the order of participant enrolment and the randomisation sequence was created by the study sponsor who was not involved in volunteer recruitment. All softgels were packed in identical containers and bottle codes were retained by the study sponsor.

### 2.3 Participants

#### 2.3.1 Inclusion criteria

Inclusion criteria comprised the following: healthy adults (female or male) aged 40–80 years; non-smoker; residing in independent living accommodation; subjective report of memory or attention problems; and had no plan to start a new intervention in the next 3 months. As the goal was to recruit generally healthy adults, participants also needed to have a body mass index (BMI) between 18 and 30 kg/m^2^, thereby excluding participants falling within the obese or underweight classifications.

#### 2.3.2 Exclusion criteria

The exclusion criteria comprised the following: a diagnosis of dementia based on the revised National Institute on Aging-Alzheimer’s Association criteria; a score below the 5th percentile for sex, age, and education on the TICS-M; a recently diagnosed or unmanaged medical condition including but not limited to diabetes, hypertension, cardiovascular disease, gastrointestinal disease, biliary disease, autoimmune disease, endocrine disease, or cancer/malignancy; a current diagnosis of a neurological condition (e.g., Parkinson’s, Alzheimer’s disease, intracranial haemorrhage, or brain injury) or psychiatric disorder (other than mild-to-moderate depression or anxiety); regular medication intake including but not limited to anticholinergics, acetylcholinesterase inhibitors, or steroid medications; a change in medication in the last 3 months or a plan to change during the study period; in the last 3 months, a change in, or the commencement of, nutritional and/or herbal supplements; the intake of herbal supplements that may influence cognitive function (e.g., nootropic herbs such as *ginkgo biloba*, ashwagandha, curcumin, *rhodiola rosea*, bacopa monnieri); alcohol consumption greater than 14 standard drinks per week; a current or 12-month history of regular illicit drug use; women who were pregnant, breastfeeding, or planned to fall pregnant during the study period; a surgery over the last year that significantly affected daily function; during the structured phone interview, a reported plan of a major lifestyle change in the next 3 months such as a significant change in diet or physical activity, relocation, extensive travel, or employment change.

### 2.4 Interventions

The intervention comprised either tocotrienols (TheraPrimE® rice) or a placebo (Safflower oil). Participants were instructed to take one softgel in the morning and one in the evening with food daily for 12 weeks. Each active softgel contained 50 mg of tocotrienols from rice brain derived from 350 mg of TheraPrimE® Rice Tocotrienol 35 Oil. The ratio of tocotrienol isomers comprises minimum specifications of 3.0% d-α-tocotrienol, 0–0.5% of d-β-tocotrienol, 8.2% of d-γ-tocotrienol, and 0.2–0.8% d-δ-tocotrienol.

In a previous study on the combined administration of tocotrienols and astaxanthin, a daily dose of 50 mg of tocotrienols was delivered for 12 weeks, with positive outcomes on cognitive function (19). Due to the hypothesised synergistic effects of the administration of the two ingredients, tocotrienols were delivered at a higher 100 mg daily dose, over an identical treatment duration of 12 weeks. Moreover, as vitamin E absorption has been shown to be enhanced by fats in a meal, intake with a meal was recommended (25).

The active and placebo softgels were identical in appearance, matched for shape, colour, and size, with both softgels containing similar excipients (safflower oil, bovine gelatin, glycerine). It should be noted that safflower oil contains a small amount of vitamin E, primarily in the form of tocopherols (26). However, the daily intake of tocopherols was estimated to be less than 0.2 mg daily, and tocotrienols was estimated to be approximately 0.005 mg daily. Adherence to capsule intake was assessed by a count of capsules returned at visit 2. Treatment blinding was evaluated by asking participants to guess their group allocation (placebo, tocotrienols, or unsure) at visit 2.

### 2.5 Outcome measures

#### 2.5.1 Primary outcome measure

*Test of Memory and Learning-2 (TOMAL-2):* The TOMAL-2 is a validated and comprehensive memory battery that provides an assessment of immediate verbal and nonverbal memory with a delayed recall component for verbal memory (27). Scores for the TOMAL-2 include the Composite Memory Index (CMI), Non-Verbal Memory Index (NVMI), Verbal Memory Index (VMI), and Verbal Delayed Recall Index (VDRI). The CMI is an overall measure of verbal and non-verbal memory and was established *a priori* as the primary outcome measure.

#### 2.5.2 Secondary outcome measures

*Behaviour Rating Inventory of Executive Function®–Adult Version (BRIEF-A):* The BRIEF-A is a validated questionnaire of executive function in adults aged 18–90 (28). Scores on the BRIEF-A include the Behavioural regulation index, Metacognition index, and the Global executive composite. The Global executive composite is the sum of the previous two scores.

*Changes in concentrations of biomarkers:* (1) Vitamin E comprising the combination of tocopherols and tocotrienol (tocotrienols could not be specifically measured with the utilised Enzyme-Linked Immunosorbent Assay); (2) Tumour Necrosis Factor-alpha (TNF-α) which is an inflammatory cytokine produced by macrophages/monocytes during acute inflammation and is responsible for a diverse range of signalling events within cells, leading to necrosis or apoptosis (29); (3) Interleukin-6 (IL-6) which is rapidly and transiently produced in response to infections and tissue injuries and contributes to host defence through the stimulation of acute phase responses, haematopoiesis, and immune reaction (30); (4) High-sensitivity C-reactive protein (hs-CRP) is a protein that rises in response to inflammation. In several studies, concentrations of hs-CRP are high in people with cognitive impairment (31, 32); (5) Malondialdehyde (MDA) is a secondary by-product of cellular lipid peroxidation of polyunsaturated fatty acids and is often used as a biomarker of oxidative stress (33); (6) Brain-derived neurotrophic factor (BDNF) plays an important role in learning, memory, and neuronal survival and growth. Disturbances in BDNF have been linked with Alzheimer’s disease and cognitive impairment (34); (7) Insulin-like growth factor 1 (IGF-1) is an essential neurotrophic factor produced peripherally and in the brain. Peripheral levels of IGF-1, measured in the serum, have been previously associated with cognitive function (35); (8) Blood lipids comprising total cholesterol, low-density lipoprotein cholesterol (LDL), high-density lipoprotein cholesterol (HDL), and triglyceride. Vitamin E supplementation has been shown to lower blood lipids (36).

*PROMIS Sleep Disturbance and Sleep-Related Impairment Scale (PROMIS Sleep):* The PROMIS Sleep is a validated 16-item self-report inventory that measures sleep quality and sleep-related impairment over the last week (37). The PROMIS Sleep is also able to significantly differentiate individuals with and without self-reported sleep disorders and between those with treated and untreated sleep disorders (37).

#### 2.5.3 Safety and expectancy measures

As expectancies can have a significant effect on outcomes in placebo-controlled studies, the Clinical Trials Treatment Expectancies Scale (CTTES) was completed by participants at visit 1. The CTTES, a 6-item questionnaire, is a revision of the Stanford Expectations of Treatment Scale (38), with wording modified to reference clinical trials on memory.

The tolerability of IP intake was assessed every month through an online question enquiring about the occurrence of any adverse events (AE). Moreover, at visit 2, participants completed the Patient Global Assessment of Tolerability to Therapy (PGATT), where they indicated their tolerability to capsule intake ranging from poor to excellent. Changes in liver function (Aspartate transaminase, Alanine aminotransferase, Alkaline Phosphatase, Gamma-glutamyl Transferase, Total Protein, Globulin, Albumin, Bilirubin), blood pressure and weight were also measured over time.

### 2.6 Sample size calculations

High-quality randomised, double-blind, placebo-controlled studies using tocotrienols as a stand-alone intervention on cognitive function in healthy adults have not been conducted. Moreover, there have been no trials using the TOMAL-2 as a primary outcome measure in nutritional trials. However, in previous trials investigating the cognitive-enhancing effects of herbal ingredients and nutraceuticals in healthy adults, effect sizes of 0.6 have been identified (39, 40). Therefore, an effect size of 0.6 was predicted (based on a single outcome variable). Based on a power of 80% and a type one (alpha) error rate of 5%, the number of total participants required to find an effect was 72. Assuming a 10–15% dropout rate, it was planned to recruit 90 participants, which was hypothesised to give suitable power to find an effect compared to the placebo, even after dropouts.

### 2.7 Statistical analysis

Outcome analyses were conducted on the full analysis set (FAS) using an intention-to-treat analysis, and on the per-protocol set (PPS), with all participants retained in originally allocated groups. FAS represents the subgroup of participants who were randomised, consumed at least one dose of the investigational product (IP), and had available efficacy data. PPS was defined as the subgroup of participants who were randomised, who consumed at least one dose of the trial product, had available efficacy data, and had no major protocol deviations (e.g., withdrew from the study, started prohibited concomitant medications, and/or completed assessments outside proposed time windows).

Generalised Linear Mixed Models (GLMM) assessed differences between intervention groups for treatment outcomes comprising the TOMAL-2 indexes, BRIEF-2 scores, PROMIS Sleep scores, and blood marker outcome measures. To examine between-group differences, changes in scores from week 0 to week 12 were calculated and GLMM were used to examine between-group differences in these scores. The covariates age, sex, BMI, and corresponding baseline scores were included in the TOMAL-2 and blood analyses, and the CTTES was included as an additional covariate for the self-reported outcome measures comprising the BRIEF-2 and PROMIS Sleep. For all GLMMs, normal (with log or identity link function) target distributions were used. Cohen’s D effect sizes were calculated for the TOMAL-2 indexes and self-report measures.

To examine within-group changes over time, GLMM was used to examine change from week 0 to week 12. The covariates age, sex and BMI were included in the TOMAL-2 and blood analyses, and the CTTES was included as an additional covariate for the self-reported outcome measures comprising the BRIEF-2 and PROMIS Sleep. For all GLMMs, normal (with log link function) target distributions were used. Appropriate covariance structures were used to model correlation associated with repeated time measurements. For safety blood measures, an independent-sample T-test was used to examine between-group differences in change in blood concentrations from week 0 to week 12, and a paired-sample T-test was used to examine within-group changes in blood concentrations over time. All data were analysed using SPSS (version 30; IBM, Armonk, NY, USA) and as there was only one *a priori* primary outcome measure, the critical *p*-value was set at *p* ≤ 0.05 for all analyses. As there was only one a priori primary outcome measure, there was no adjustment to the *p*-value for multiple testing.

## 3 Results

### 3.1 Study population

A total of 131 people were screened by telephone and 91 people were randomised. The most common grounds for exclusion were withdrawing consent after the telephone interview (*n* = 22) and engaging in extensive travel during the trial (*n* = 10). Baseline demographic and clinical characteristics are detailed in Table 1. The two groups were matched similarly in age, BMI, marital status, educational level, and sex distribution. Baseline scores on outcome measures were also similar between the two groups.

**Table 1:** Baseline sociodemographic and clinical characteristics.

Sociodemographic detail and outcome baseline measures		Placebo (*N* = 45)	Tocotrienols (*N* = 46)
Age (yrs)	Mean	63.29	66.04
	SE	1.80	1.56
Sex (n)	Male	17	11
	Female	28	35
Height (m)	Mean	1.69	1.64
	SE	0.01	0.01
Weight (kg)	Mean	75.50	69.70
	SE	1.81	1.54
BMI (kg/m^2^)	Mean	26.18	25.68
	SE	0.40	0.39
TICS percentile	Mean	54.68	57.90
	SE	3.82	4.34
Systolic blood pressure (mmHg)	Mean	135.40	132.48
	SE	3.05	2.63
Diastolic blood pressure (mmHg)	Mean	81.18	78.65
	SE	1.67	1.01
Marital Status (n)	Single	21	14
	Married/defacto	24	32
Education (n)	Secondary	25	23
	Tertiary	15	15
	Post-graduate	5	8
International Physical Activity Questionnaire category (n)	Low	14	17
	Moderate	25	22
	High	6	7
Occupation (n)	Retired	22	25
	Technicians and associated trades	10	5
	Professional	5	5
	Unemployed	1	2
	Services and sales worker	2	5
	Manager	4	0
	Elementary occupation	1	2
	Clerical support worker	0	2
TOMAL-2 CMI (raw score)	Mean	89.91	90.63
	SE	2.082	1.864
TOMAL-2 VMI (raw score)	Mean	94.42	97.17
	SE	2.092	1.993
TOMAL-2 NVMI (raw score)	Mean	88.36	86.83
	SE	1.992	1.755
TOMAL-2 VDRI (raw score)	Mean	85.62	91.28
	SE	2.232	2.175
BRIEF-2 Global Executive Composite (T-score)	Mean	60.56	60.20
	SE	1.64	1.30
PROMIS Sleep Disturbance T-Score	Mean	49.83	51.37
	SE	1.11	1.02
PROMIS Sleep-Related Impairment T-Score	Mean	49.79	48.48
	SE	1.31	1.11

BMI, Body Mass Index; CMI, Composite memory index; NVMI, Non-verbal memory index; SE, Standard Error; TICS-M, Telephone Interview for Cognitive Status – Modified Version; TOMAL-2, Test of Memory and Learning version 2; VMI, Verbal memory index; VDRI, Verbal delayed recall index.

### 3.2 Outcome measures

**Figure 2:**
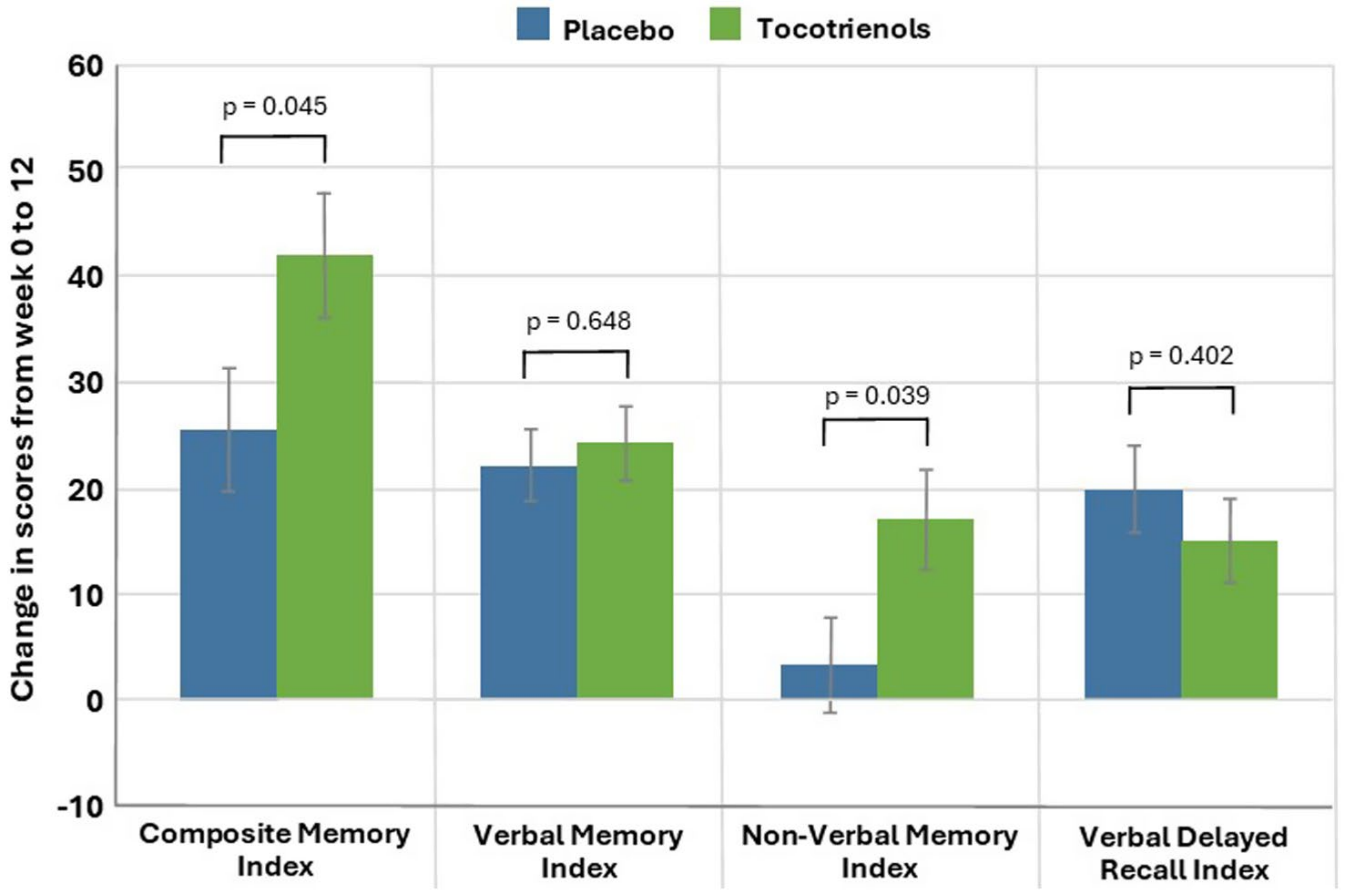
Change in TOMAL-2 Index Scores from Baseline to Week 12 (Error bars represent standard errors).

*TOMAL-2 scores:* As demonstrated in Table 2 and Figure 2, based on the GLMM, the CMI (primary outcome measure) significantly increased from baseline to week 12 in both the tocotrienols and placebo groups (*p* < 0.001). After adjustment for baseline values, CMI increased by a mean of 41.93 points (95% CI: 30.23, 53.63) in the tocotrienols group and increased by 25.65 points (95% CI: 41.40, 36.90) in the placebo group. These changes were significantly different between the two groups (*p* = 0.045, ES = 0.46, 95% CI: 0.34, 32.21). An analysis of the PPS revealed similar, statistically significant between-group differences (*p* = 0.043, ES = 0.47, 95% CI: 0.51, 31.39) (Supplementary Table 1).

**Table 2:** Change in TOMAL-2 scores (estimated marginal means) (Full analysis set).

TOMAL-2 scores		Placebo (*n* = 45)				Tocotrienols (*n* = 46)				*p*-value^b^	Cohen’s D
		Week 0	Week 12	Change^b^	*p*-value^a^	Week 0	Week 12	Change^b^	*p*-value^a^		
TOMAL-2: CMI (sum of raw scores)	Mean	243.73	259.58	25.65	<0.001	245.41	266.29	41.93	<0.001	0.045	0.46
	SE	5.28	5.35	5.74		5.54	5.65	5.97			
TOMAL-2: VMI (sum of raw scores)	Mean	258.42	282.58	22.24	<0.001	264.72	288.79	24.38	<0.001	0.648	0.10
	SE	6.16	6.27	3.38		6.44	6.59	3.47			
TOMAL-2: NVMI (sum of raw scores)	Mean	238.30	242.28	3.36	0.404	232.24	252.68	17.01	<0.001	0.039	0.47
	SE	7.10	7.21	4.66		7.47	7.64	4.85			
TOMAL-2: VDRI (sum of raw scores)	Mean	23.06	28.22	19.78	<0.001	25.84	30.46	14.85	<0.001	0.402	0.19
	SE	1.26	1.30	4.21		1.32	1.38	4.26			

Results (estimated means) are generated from generalised mixed-effects models adjusted for age, sex, and BMI. ^a^*p*-values are generated from repeated measures generalised mixed-effects models adjusted for age, sex, and BMI. ^b^*p*-values and change scores are generated from the change in mean scores from week 0 using generalised mixed-effects models adjusted for age, sex, BMI, and corresponding baseline scores.

Scores on the NVMI significantly increased from baseline to week 12 in the tocotrienols group (*p* < 0.001), but not in the placebo group (*p* = 0.404). After adjustment for baseline values, NVMI increased by a mean of 17.01 points (95% CI: 7.50, 26.52) in the tocotrienols group and non-significantly increased by 3.36 points (95% CI: −5.77, 12.49) in the placebo group. These changes were significantly different between the two groups (*p* = 0.039, ES = 0.47, 95% CI: 0.68, 26.63). An analysis of the PPS revealed similar, statistically significant between-group differences (*p* = 0.048, ES = 0.46, 95% CI: 0.15, 26.10) (Supplementary Table 1).

VMI scores significantly increased from baseline to week 12 in both the tocotrienols and placebo groups (*p* < 0.001). However, these changes were not significantly different between the two groups (*p* = 0.648, 95% CI: −7.16, 11.44). An analysis of the PPS revealed similar, non-significant between-group differences (*p* = 0.612, 95% CI: −7.08, 11.94) (Supplementary Table 1). Moreover, there were no between-group differences in changes in the VDRI.

An exploratory analysis was conducted to determine if changes in TOMAL-2 scores differed based on age, categorised as middle age (40–59 years) and older age (≥60 years). This revealed that Cohen’s D effect sizes for change in TOMAL-2 scores were generally equivalent, although it was slightly higher for changes in NVMI in the older age group, where the Cohen’s D effect size was 0.56 compared to 0.38 in the middle-age group. However, given the small sample sizes, this difference should be viewed tentatively. Descriptive statistics are detailed in Supplementary Table 2.

*BRIEF-A:* As demonstrated in Table 3, based on the GLMM, there were no between-group differences in changes in any BRIEF-A scores over time.

**Table 3:** Change in self-report questionnaires from week 0 to week 12 (estimated marginal means) (Full analysis set).

Self-report measures		Placebo (*n* = 45)						Tocotrienols (*n* = 46)						*p*-value^b^	Cohen’s D
		Week 0	Week 4	Week 8	Week 12	Change^b^	*p*-value^a^	Week 0	Week 4	Week 8	Week 12	Change^b^	*p*-value^a^		
BRIEF-2: BRI (T-score)	Mean	57.40	–	–	55.70	−1.88	0.072	57.25	–	–	55.88	−1.28	0.133	0.651	0.10
	SE	1.50	–	–	1.51	0.92		1.54	–	–	1.55	0.97			
BRIEF-2: MI (T-score)	Mean	61.49	–	–	60.20	−1.29	0.134	59.26	–	–	56.66	−2.88	0.002	0.200	0.29
	SE	1.53	–	–	1.54	0.86		1.56	–	–	1.56	0.91			
BRIEF-2: GEC (T-score)	Mean	60.24	–	–	58.83	−1.48	0.088	58.97	–	–	56.65	−2.44	0.004	0.414	0.19
	SE	1.46	–	–	1.47	0.82		1.50	–	–	1.50	0.87			
PROMIS Sleep Disturbance (T-score)	Mean	49.47	48.95	49.08	48.34	−1.73	0.170	49.84	48.94	49.16	46.08	−4.08	<0.001	0.036	0.48
	SE	1.07	1.08	1.08	1.08	0.77		1.10	1.10	1.10	1.09	0.81			
PROMIS Sleep-Related Impairment (T-score)	Mean	49.71	48.46	48.37	47.65	−1.99	0.054	47.17	45.99	47.19	44.12	−4.01	0.003	0.202	0.29
	SE	1.33	1.34	1.34	1.35	1.09		1.35	1.36	1.37	1.35	1.16			

Results (estimated means) are generated from generalised mixed-effects models adjusted for age, sex, BMI, and CTTES positive and negative scores. ^a^*p*-values are generated from repeated measures generalised mixed-effects models adjusted for age, sex, BMI, and CTTES positive and negative scores. ^b^*p*-values and change scores are generated from the change in mean scores from week 0 to week 12 using generalised mixed-effects models adjusted for age, sex, BMI, CTTES positive and negative scores, and corresponding baseline scores.

*Blood Biomarkers:* As demonstrated in Table 4, there were statistically significant between-group differences in changes in TNF-α (*p* = 0.043) and hs-CRP (*p* = 0.039). In relation to TNF-α, concentrations increased significantly in the placebo group (*p* < 0.001), and there was a non-significant increase in the tocotrienols group (*p* = 0.071). In relation to hs-CRP, concentrations increased significantly in the tocotrienols group (*p* = 0.041), and there was a non-significant decrease in the placebo group (*p* = 0.177). There were no statistically significant between-group differences in changes in other blood markers comprising MDA (*p* = 0.730), vitamin E (*p* = 0.858), IL-6 (*p* = 0.139), IGF (*p* = 0.717), BDNF (*p* = 0.216), and blood lipids comprising total cholesterol (*p* = 0.500), HDL (*p* = 0.719), LDL (*p* = 0.602), and triglyceride (*p* = 0.659). However, an examination of within-group changes over time revealed a statistically significant decrease in MDA in the tocotrienols group (*p* = 0.033) and a statistically significant increase in IL-6 in the placebo group (*p* = 0.005).

**Table 4:** Change in blood concentrations over time (Full analysis set).

Blood measures		Placebo				Tocotrienols				*p*-value^b^
		Week 0	Week 12	Change^b^	*p*-value^a^	Week 0	Week 12	Change^b^	*p*-value^a^	
MDA (ng/mL)	Mean	78.4	69.21	−12.57	0.092	88.91	77.5	−10.38	0.033	0.73
	SE	5.58	5.6	4.44		5.78	5.65	4.55		
	N	37	37	37		39	39	39		
Vitamin E (mg/mL)	Mean	6.95	7.34	0.35	0.449	8.56	9.29	0.48	0.152	0.858
	SE	1.46	1.47	0.53		1.56	1.59	0.54		
	N	37	37	37		39	39	39		
IL-6 (pg/mL)	Mean	38.23	43.57	5.38	0.005	37.79	39.06	1.11	0.446	0.139
	SE	6.26	6.39	2.03		6.11	6.16	2.08		
	N	38	38	38		40	40	40		
TNF-α (pg/mL)	Mean	34.84	43.66	8.92	<0.001	29.93	33.14	3.55	0.071	0.043
	SE	3.74	3.75	1.86		3.75	3.78	1.89		
	N	38	38	38		40	40	40		
IGF (ng/mL)	Mean	162.67	154.73	−3.32	0.236	144.82	148.15	0.02	0.619	0.717
	SE	7.88	7.87	6.53		8.01	8.03	6.59		
	N	38	38	38		40	40	40		
BDNF (pg/mL)	Mean	624.05	616.22	−20.83	0.886	716.33	762.6	62.97	0.400	0.216
	SE	56.02	55.99	47.35		57.61	57.92	48.85		
	N	38	38	38		40	40	40		
hs-CRP (mg/L)	Mean	2.18	1.58	−0.57	0.177	1.40	2.28	0.86	0.041	0.039
	SE	0.46	0.44	0.48		0.41	0.46	0.5		
	N	35	35	35		44	44	44		

Results (estimated means) are generated from generalised mixed-effects models adjusted for age, sex, and BMI. ^a^*p*-values are generated from repeated measures generalised mixed-effects models adjusted for age, sex, and BMI. ^b^*p*-values and change scores are generated from the change in mean scores from week 0 using generalised mixed-effects models adjusted for age, sex, BMI, and corresponding baseline score.

*PROMIS Sleep:* As demonstrated in Table 3, the sleep disturbance T-score significantly decreased from baseline to week 12 in the tocotrienols group (*p* < 0.001) but not in the placebo group (*p* = 0.170). These changes were significantly different between the two groups (*p* = 0.036, ES = 0.48, 95% CI: −0.15, −4.53). An analysis of the PPS revealed similar, statistically significant between-group differences (*p* = 0.015, ES = 0.58, 95% CI: −4.80, −0.55) (Supplementary Table 2). The sleep-related impairment T-score significantly decreased from baseline to week 12 in the tocotrienols group (*p* = 0.003) but not in the placebo group (*p* = 0.054). However, these changes were not significantly different between the two groups (*p* = 0.202, 95% CI: −5.16, 1.11). An analysis of the PPS revealed similar, non-significant between-group differences (*p* = 0.179, 95% CI: −5.38, −1.02) (Supplementary Table 3).

An exploratory mediation analysis was conducted to determine if changes in cognitive performance were mediated by changes in sleep quality. A linear regression indicated that neither changes in sleep disturbances (*p* = 0.490) nor sleep impairment (*p* = 0.875) were statistically significant mediators of change in the TOMAL-2 CMI. Similar non-significant findings were found for changes in the TOMAL-2 NVMI.

### 3.3 Intake of supplements

IP bottles with remaining softgels were counted by investigators at visit 2. Based on these details, all participants who completed the study took over 80% of their capsules.

### 3.4 Adverse reactions and treatment discontinuation

Participants reported no serious AEs, and there was a similar frequency of AEs classified as possibly or probably related to the IP intake (Supplementary Table 5). In the placebo group, 4.5% (*n* = 2) of participants experienced a treatment-related AE while in the tocotrienols group, 6.5% (*n* = 3) of participants experienced a treatment-related AE. The PGATT results are detailed in Supplementary Table 6, which demonstrates that in both groups over 95% of participants reported good or excellent tolerability to the IP. Two people (4.5%) in the tocotrienols group reported moderate tolerability, and in the placebo group, 100% of participants who completed the study reported good or excellent tolerability to the IP. A total of 5 people discontinued the study, comprising two in the tocotrienols group and 3 in the placebo group. In the tocotrienols group, reasons provided for study discontinuation included skin itchiness (*n* = 1), and unexpected travel (*n* = 1). An analysis of liver function markers revealed no statistically significant between-group differences in changes in any markers over time. It is important to note that at week 12, 5 participants in the tocotrienols group and 1 participant in the placebo group had an elevated hs-CRP (>5 mg/L) that could not be attributed to illnesses, changes in medications, other factors that are associated with elevations in hs-CRP. Therefore, these were classified as adverse events possibly related to the IP. However, there was significant variability in hs-CRP as 10 participants had hs-CRP concentrations above 5 mg/L at baseline. An analysis of participants in the tocotrienols group who experienced increases in hs-CRP at week 12 that were classified as possibly related to the IP, revealed that 4 out of 5 were female, and all of them experienced a reduction in blood concentration of vitamin E over time. These reductions in vitamin E could not be attributed to inconsistent IP intake as adherence in these participants was greater than 90%. Age and weight fluctuations in these participants could not account for hs-CRP increases.

As detailed in Supplementary Table 7, there were no between-group or within-group changes over time in BMI or diastolic blood pressure. However, there was a statistically significant between-group difference in changes in systolic blood pressure over time (*p* = 0.021). In the tocotrienols group, systolic blood pressure was reduced by 5.14 mmHg (*p* = 0.011) and there was no statistically significant change in the placebo group.

### 3.5 Efficacy of participant blinding

To assess the effectiveness of condition concealment during the trial, participants predicted their condition allocation (i.e., placebo, tocotrienols, or unsure) at the end of the study. Overall group concealment was high, as only 32% of participants correctly guessed treatment allocation.

## 4 Discussion

In this randomised, double-blind, placebo-controlled study, the effects of daily supplementation with 100 mg of tocotrienols (TheraPrimE® rice) for 12 weeks in adults aged 40–80 years with SMC were examined. Based on the primary outcome measure, supplementation with tocotrienols was associated with a greater improvement in overall memory (TOMAL-2 CMI) compared to the placebo. In the tocotrienols group, CMI scores increased by 8.3% compared to a smaller 5.1% increase in the placebo group. A more detailed examination of memory changes revealed that improvements in memory primarily arose from enhancements in non-verbal memory. In the tocotrienols group, non-verbal memory scores increased by 7.1% in the tocotrienols group, whereas scores non-significantly increased by only 1.4% in the placebo group. No group differences in changes in verbal memory were observed, which may be partly accounted for by statistically significant improvements in both groups over time. Moreover, there were no group differences in changes in self-reported executive function (BRIEF-2). However, in the tocotrienols group, there were significantly greater improvements in self-reported sleep disturbance compared to the placebo group, as evidenced by an 8.1 and 3.4% reduction, respectively. To examine the potential mechanisms of action associated with tocotrienol supplementation, blood markers associated with inflammation (TNF-α, hs-CRP, and IL-6), oxidative stress (MDA), and neurotrophic activity (BDNF and IGF-1) were measured over time. Group differences in changes in hs-CRP and TNF-α were observed. The group differences in hs-CRP were the result of a statistically significant increase in hs-CRP in the tocotrienols group and a non-significant decrease in the placebo group over time. In relation to TNF-α, there was a statistically significant increase in the placebo group and a non-significant increase in the tocotrienols group. No group differences in changes in the other blood markers were identified. Finally, changes in blood concentrations of vitamin E were measured over time. This revealed no statistically significant changes over time in either group. However, it is important to note that the assay used could not specifically measure tocotrienols but instead measured a combination of tocopherols and tocotrienols. In the placebo group, vitamin E concentrations non-significantly increased by 4.0%, and in the tocotrienols group, there was a near statistically significant increase of 7.2%. Tocotrienol supplementation was well tolerated with no serious adverse reactions and a similar frequency of AEs in the placebo and tocotrienols group. Moreover, there were no changes in weight and diastolic blood pressure over time. However, tocotrienol supplementation was associated with a greater reduction in systolic blood pressure compared to the placebo group, where it reduced by a mean of 5.14 mmHg compared to a 1.38 mmHg decrease in the placebo group.

This is the first controlled clinical trial investigating the effects of tocotrienols as a stand-alone intervention on cognitive function in healthy adults. However, there is evidence from cross-sectional and longitudinal studies that higher vitamin E intake and higher blood concentration of vitamin E are associated with better cognitive function and a reduced prevalence of AD and cognitive decline. In a meta-analysis of 31 studies, it was concluded that individuals with AD or age-related cognitive decline had lower circulatory concentrations of α-tocopherol compared with healthy controls (11). In relation to investigations specifically on tocotrienols, a prospective investigation over a 6-year period demonstrated that in adults aged over 80 years, a higher blood tocotrienol concentration was associated with a reduced risk of developing AD (41). In another prospective study conducted over 8 years, individuals who developed cognitive impairment had significantly lower blood concentrations of β- and γ-tocotrienols (42). However, after adjustment for multiple confounders, significance only remained for γ-tocotrienols.

Findings from vitamin E interventional trials on cognitive function have demonstrated inconsistent results. In a review of four trials comprising 304 adults with AD and 516 adults with MCI, it was concluded that there was no evidence that vitamin E in the form of α-tocopherol given to people with MCI prevented progression to dementia, or improved cognitive function in people with MCI or AD. However, there was moderate-quality evidence from a single study that it may slow functional decline in AD (12). In this randomised, double-blind clinical trial in patients with mild to moderate AD taking an acetylcholinesterase inhibitor, 2,000 international units (IUs) a day of α-tocopherol for a mean duration of 2.3 years, resulted in slower functional decline compared to the placebo (43). Interestingly, there were no significant differences in the groups receiving memantine alone or memantine plus α-tocopherol. However, based on the findings of the Prevention of Alzheimer’s Disease by Vitamin E and Selenium (PREADViSE) trial, men aged 60 years and older did not obtain any benefit from vitamin E supplementation (44).

As a stand-alone intervention, there has been limited investigation into the effects of tocotrienols on cognitive function. One randomised, double-blind, placebo-controlled study was identified where children aged 6–12 years with ADHD were supplemented with 200 mg of tocotrienols daily for 6 months (18). Tocotrienols had no significant effect on a parent-rated ADHD measure, but there was a near-statistically significant group difference in the teacher version. At the end of the study, higher α- and γ-tocotrienol levels were also positively correlated with changes in the total symptom score on the parent version of the ADHD outcome measure.

Investigations in non-clinical populations have demonstrated that when administered in combination with other nutrients, vitamin E may be beneficial to cognitive function. In a 12-week study on healthy adults, the administration of tocotrienols with astaxanthin resulted in significantly greater improvements in composite memory and verbal memory compared to the placebo group (19). In another study on healthy adults aged 40–70 years, the combination of α-tocopherol, grape juice extract, and astaxanthin for 12 weeks was associated with greater improvements in episodic memory compared to the placebo (45). A larger increase in plasma BDNF and a decrease in MDA was also observed in the supplemented group compared to the placebo. In another study, improvements in working memory were also observed after 24 months of supplementation with a combination of vitamin E, fish oil, and carotenoids in cognitively healthy adults aged 65 years and older (46). However, when vitamin E was administered with vitamin C for 12 months to adults aged 60–75 years with MCI, there were no group differences in cognitive performance compared to the placebo, despite significant reductions in MDA and increases in total antioxidant capacity and glutathione (47). In summary, the results from the current study, along with results from previous nutraceutical combination trials, suggest supplementation with vitamin E, particularly tocotrienols, may be beneficial to cognitive function in healthy adults with SMC. However, its administration seems less efficacious in people with AD and MCI.

How vitamin E improves cognitive function, specifically non-verbal memory, requires further investigation. The right brain hemisphere, and in particular the right hippocampus, has important influences on non-verbal memory (48). Neuroinflammation and reduced antioxidant capacity can negatively affect adult hippocampal neurogenesis and cognition (49, 50). Based on preclinical and clinical data, vitamin E may have neuroprotective effects through its effects on β-amyloid plaque accumulation, tau-protein hyperphosphorylation, antioxidant and anti-inflammatory properties, and positive effect on metabolic functioning of mitochondria (51–54). There is also research demonstrating it may increase neurotrophic activity by increasing BDNF concentrations in brain regions such as the hippocampus (52, 55, 56). However, these mechanisms were not demonstrated in this study, with no group differences in changes in plasma BDNF or IGF. Moreover, there were no group differences in changes in MDA, a marker of oxidative stress, although in the tocotrienols group, there was a statistically significant decrease in MDA over time. In relation to the anti-inflammatory effects of tocotrienols, there were inconsistent findings. There were no group differences in changes in IL-6 over time, although in the placebo group, IL-6 significantly increased over time. In relation to TNF-α, group differences were demonstrated, primarily due to a larger increase in the placebo group over time. In contrast to these findings, tocotrienol supplementation was associated with a small (0.86 mg/L), but statistically significant increase in hs-CRP over time, compared to the placebo group. This increase in hs-CRP could not be accounted for by changes in medications, weight, or illnesses. This finding requires examination in future clinical trials as a meta-analysis based on 13 studies revealed that tocotrienol supplementation was associated with reductions in CRP concentrations (57). Interestingly, Del Guidice and Gangestad (58) have reported that CRP has two isoforms, one of which is produced locally in inflamed or damaged tissues, while the other is routinely produced in the absence of inflammation and may have net anti-inflammatory effects. They report that while very high concentrations of CRP likely indicate acute inflammation, moderate levels of circulating CRP may not indicate the presence of low-grade chronic inflammation, but rather, it may be due to its involvement in wound healing, tissue repair, and the clearance of damaged cells. The two CRP isoforms were not differentially measured in this study. It is also important to note that the increase in hsCRP over time in the tocotrienols group was unlikely to have clinical relevance as concentrations continued to remain within normal thresholds (2.28 mg/L). Interestingly, the mean concentration of hsCRP at week 12 in the tocotrienols group was similar to the mean concentration at baseline (2.18 mg/L) in the placebo group, thereby providing further confirmation of the likely normal variation that occurs over time.

A unique exploratory finding from this study related to improvements in self-reported sleep disturbance in participants supplemented with tocotrienols compared to the placebo group. While further research is required to substantiate this finding, this has important implications as sleep disturbances are associated with an increased risk of mental and physical diseases, including an increased risk of cognitive decline and AD (59, 60). Investigations into the sleep-enhancing effects of vitamin E are limited, although in one randomised, double-blind, placebo-controlled trial in postmenopausal women with chronic insomnia, 400 IUs of mixed tocopherols daily for one month was associated with better sleep quality and a reduction in the use of sedative drugs (61). In an animal trial, the chronic administration of vitamin E ameliorated impairments in both short and long-term memory after chronic sleep deprivation. Vitamin E also normalised sleep deprivation-induced reductions in hippocampus glutathione/oxidised glutathione ratio; and catalase, superoxide dismutase, and glutathione peroxidase activity (62). Inflammation and oxidative stress are associated with insomnia and sleep disturbances, and vitamin E may provide sleep-related benefits via these mechanisms (21, 22). As insomnia and poor sleep are often associated with poorer cognitive function (20), an analysis was undertaken to determine if changes in cognitive performance may be mediated by changes in sleep quality. However, this was not demonstrated in this study, suggesting that other factors likely accounted for the improvements in cognitive function.

### 4.1 Strengths, limitations, and directions for future research

The strengths of this study include the randomised, double-blind, placebo-controlled design, where validated objective measures of memory and subjective measures of executive function and sleep were administered. However, the recruitment of healthy adults with SMC and possible ceiling effects on many subtests may have impacted the ability to sensitively detect changes in memory and other symptomatic changes over time. Improvements in verbal memory but not non-verbal memory occurred in the placebo group, which may partly account for the non-significant group differences in changes in verbal memory over time. This suggests there were greater practice effects on the verbal memory subtest, with no such overall effects occurring in tests of non-verbal memory. It is also important to note that the recruitment of participants with digital and social media access may limit the generalisability of the findings. The assessment of psychological status using only a brief self-report scale, and the TICS-M to screen for cognitive impairment at baseline, may impact on the sensitivity to detect undiagnosed cognitive and psychological impairment.

The examination of changes in blood measures over time helped to determine the mechanisms of action associated with tocotrienols supplementation. However, significant variability in hsCRP concentrations and measurement of total vitamin E concentrations using ELISA impacted the strength of conclusions obtained in the study. Investigation of changes in blood concentrations of tocotrienols and various isoforms will be important in future studies to examine the effects of supplementation on body stores; and the relationship between changes in total tocotrienols and its isoforms on changes in cognitive function. This should be undertaken using validated High Performance Liquid Chromatography and liquid chromatography-mass spectrometry methods. Therefore, the lack of change in blood vitamin E concentration identified in this study should be considered speculative. Moreover, the generally non-significant group differences in changes in markers of inflammation, oxidative stress, and neurotrophic activity may be partly attributed to the population recruited for this study. That is, generally healthy, non-obese adults with no medical diseases or having a well-managed medical condition. This may result in floor or ceiling effects, where further reductions/increases would be unlikely. This is demonstrated by an overall mean hsCRP concentration of 1.9 mg/L at baseline, which is well within normal concentrations (63). Therefore, to help further examine the effects of tocotrienols on inflammation, oxidative stress, and neurotrophic activity, the populations who are more likely to exhibit disturbances in these areas should be recruited.

The following recommendations are provided for future research. As this study investigated the effects of tocotrienols, a comparative examination into the efficacy and safety of supplementation with tocotrienols versus tocopherols will be useful. Moreover, as tocotrienols have 4 isoforms, a further examination of differences in the efficacy of different isoforms may be useful. It will also be important to determine if greater outcomes are achieved when isoforms are delivered in combination or as single isoforms. The effects of tocotrienols derived from annatto, rice, or palm oils will also be important to investigate.

In this study, non-verbal memory improved after supplementation, but further investigation into the effects of tocotrienols on other cognitive domains will be important. This includes a further examination of its effects on episodic memory, working memory, attention, and concentration.

In this study, tocotrienols supplementation was well tolerated with no serious adverse events reported, and good to excellent tolerability was reported by most participants. This is particularly encouraging as approximately 70% of participants were aged over 60 years. While some therapeutic efficacy was demonstrated with stand-alone tocotrienol supplementation at 100 mg daily, examining the efficacy and safety of supplementation at varying doses, with or without other nutritional ingredients, will be important. Longer interventions will also be worthwhile, as more than 3 months will likely be required for neurological changes to occur. A longer study will also help to determine if tocotrienol supplementation can prevent the progression from SMC to MCI or AD. Future trials should also be undertaken to examine the mechanisms of action of tocotrienol supplementation, particularly in relation to its impact on cognitive performance. It is possible that the effects of tocotrienol supplementation may have greater efficacy in people experiencing increased oxidative stress and/or inflammation. This includes older age adults, people eating unhealthy diets or people with comorbid medical conditions, where inflammation and oxidative stress may be a major driver. An exploratory analysis indicated generally similar changes in cognitive performance in the younger cohort (40–59 years) compared to the older cohort (≥60 years); however, there was some suggestion of greater cognitive gains from tocotrienol supplementation in the older cohort on non-verbal memory. This requires further investigation due to the small sample sizes from this subgroup analysis. Finally, a further examination into the effects of tocotrienol supplementation in people experiencing sleep disturbances will help clarify the effects of tocotrienols on sleep quality. Although changes in sleep did not account for the changes in cognitive function, given the strong link between sleep and cognitive performance, this will be worthy of further investigation.

In relation to the clinical value of supplementation with tocotrienols on cognitive function, categorical yes or no conclusions without taking into account important contextual factors are advised against by the authors. Typically, conclusions on clinically meaningful changes are based on thresholds established on the outcome measures used. However, no minimal clinically important difference (MCID) thresholds were identified on the TOMAL-2 scores. An effect size of 0.46, suggesting a moderate effect size from supplementation, was identified. However, important contextual factors that need to be considered when determining the meaningfulness of change include treatment safety and tolerability, treatment costs, treatment resources, patient burden, the population examined, symptom severity, and treatment duration, just to name a few. Based on these criteria, in a population of generally healthy adults with SMC, tocotrienols supplementation for 12 weeks was generally affordable (will likely cost less than $30 USD a month), resource-light, and very well tolerated, resulting in minimally noticeable improvements in cognitive performance, particularly non-verbal memory, for participants. As a stand-alone intervention, it is unlikely to be clinically meaningful, but as an adjunct intervention to dietary, environmental, and lifestyle changes, it is up to consumers and health practitioners to determine whether its benefits outweigh its costs as an aid to support cognitive function. A detailed qualitative and quantitative evaluation of the clinical meaningfulness/importance of the current study findings, which has been developed by the first author, is included in Supplementary Table 7.

In summary, this study provides evidence of the beneficial effects of tocotrienol supplementation for 12 weeks on non-verbal memory in adults with SMC. Future trials in participants with more severe cognitive deficits and over a longer treatment period will help to substantiate the safety and efficacy of tocotrienols as a prevention or treatment for cognitive decline in adults.

## Data Availability

The raw data supporting the conclusions of this article will be made available by the authors without undue reservation.
