# Supplementary material for: An examination into the effects of tocotrienols (TheraPrimE® rice) on cognitive abilities and sleep in healthy adults: a randomised, double-blind, placebo-controlled trial
Source: Front Nutr. 2025 Sep 3;12:1621516. doi: 10.3389/fnut.2025.1621516 (PMC12440894; doi:10.3389/fnut.2025.1621516)
Supplement: Supplementary file 1 [file Table_1.DOCX]

######

Supplementary Material

**An examination into the effects of tocotrienols (TheraPrimE® rice) on cognitive abilities in healthy adults: a randomised, double-blind, placebo-controlled trial**

**Author names:**

^1,2^ Adrian L Lopresti - ORCID iD: 0000-0002-6409-7839

^1^ Stephen J Smith - ORCID iD: 0000-0002-3875-4815

^3^ Lixin Ding

^4^ Yanmei Li

^4^ Peinan Zhang

**Author affiliations:**

^1^Clinical Research Australia, Perth, Western Australia, 6023, Australia

^2^College of Science, Health, Engineering and Education, Murdoch University, Perth, Western Australia, 6150, Australia

^3^BGG Americas, 26 Thomas, Irvine, CA 92618, USA

^4^BGG, Room1006, South Block of Daheng Technology Building, No. 3 Suzhou Street, Haidian District, Beijing 100080, China

**Corresponding author:** Adrian Lopresti, Clinical Research Australia, 38 Arnisdale Road Duncraig, Western Australia 6023, [adrian@clinicalresearch.com.au](mailto:adrian@clinicalresearch.com.au)

## Supplementary Table 1. Change in TOMAL-2 scores (estimated marginal means) (Per Protocol Set)

|  | | Placebo (n=41) | | | | Tocotrienols (n=41) | | | | p-value^b^ | Cohen's D |
| --- | --- | --- | --- | --- | --- | --- | --- | --- | --- | --- | --- |
|  |  | Week 0 | Week 12 | Change^b^ | p-value^a^ | Week 0 | Week 12 | Change^b^ | p-value^a^ |  |  |
| TOMAL-2: CMI (sum of raw scores) | Mean | 494.85 | 522.28 | 25.69 | <.001 | 498.14 | 541.23 | 41.64 | <.001 | 0.043 | 0.47 |
|  | SE | 12.26 | 12.32 | 5.49 |  | 12.94 | 13.14 | 5.76 |  |  |  |
| TOMAL-2: VMI (sum of raw scores) | Mean | 256.47 | 280.42 | 22.29 | <.001 | 266.56 | 290.47 | 24.72 | <.001 | 0.612 | 0.12 |
|  | SE | 6.49 | 6.54 | 3.41 |  | 6.84 | 6.95 | 3.52 |  |  |  |
| TOMAL-2: NVMI (sum of raw scores) | Mean | 238.26 | 241.61 | 3.24 | 0.481 | 231.95 | 251.06 | 16.36 | <.001 | 0.048 | 0.46 |
|  | SE | 7.55 | 7.56 | 4.59 |  | 7.97 | 8.08 | 4.84 |  |  |  |
| TOMAL-2: VDRI (sum of raw scores) | Mean | 92.64 | 115.97 | 19.98 | < .001 | 108.04 | 122.66 | 15.27 | 0.001 | 0.436 | 0.18 |
|  | SE | 5.53 | 5.68 | 4.26 |  | 5.91 | 6.10 | 4.36 |  |  |  |

Results (estimated means) are generated from generalised mixed-effects models adjusted for age, sex, and BMI. ^a^P-values are generated from repeated measures generalised mixed-effects models adjusted for age, sex, and BMI. ^b^P-values and change scores are generated from the change in mean scores from week 0 using generalised mixed-effects models adjusted for age, sex, BMI, and corresponding baseline scores.

## Supplementary Table 2. Change in TOMAL-2 scores based on age category (estimated marginal means) (Full Protocol Set)

|  |  | Age 40 to 59 years | | | | | | | Age ≥ 60 years | | | | | | |
| --- | --- | --- | --- | --- | --- | --- | --- | --- | --- | --- | --- | --- | --- | --- | --- |
|  |  | N | Mean | SE | 95% Confidence Interval | | p-value | Cohen's D | N | Mean | SE | 95% Confidence Interval | | p-value | Cohen's D |
|  |  |  |  |  | Lower | Upper |  |  |  |  |  | Lower | Upper |  |  |
| Change in TOMAL-2: CMI (sum of raw scores) | Placebo | 18 | 36.65 | 7.45 | 21.31 | 51.99 | 0.301 | 0.425 | 24 | 19.63 | 8.48 | 2.60 | 36.65 | 0.109 | 0.458 |
|  | Tocotrienols | 12 | 50.42 | 10.62 | 28.55 | 72.28 |  |  | 32 | 36.80 | 7.23 | 22.29 | 51.31 |  |  |
| Change in TOMAL-2: VMI (sum of raw scores) | Placebo | 18 | 28.21 | 3.90 | 20.19 | 36.24 | 0.753 | 0.128 | 24 | 20.21 | 4.95 | 10.26 | 30.16 | 0.857 | 0.051 |
|  | Tocotrienols | 12 | 30.38 | 5.55 | 18.94 | 41.81 |  |  | 32 | 21.31 | 4.13 | 13.01 | 29.60 |  |  |
| Change in TOMAL-2: NVMI (sum of raw scores) | Placebo | 18 | 7.79 | 7.35 | -7.34 | 22.92 | 0.352 | 0.382 | 24 | -0.77 | 6.38 | -13.58 | 12.04 | 0.052 | 0.559 |
|  | Tocotrienols | 12 | 19.98 | 10.38 | -1.41 | 41.36 |  |  | 32 | 15.22 | 5.57 | 4.03 | 26.40 |  |  |
| Change in TOMAL-2: VDRI (sum of raw scores) | Placebo | 18 | 18.10 | 5.10 | 7.59 | 28.60 | 0.649 | 0.185 | 24 | 20.91 | 6.42 | 8.02 | 33.80 | 0.313 | 0.286 |
|  | Tocotrienols | 12 | 22.19 | 7.22 | 7.32 | 37.05 |  |  | 32 | 12.72 | 5.31 | 2.07 | 23.38 |  |  |

P-values and change scores are generated from the change in mean scores from week 0 using generalised mixed-effects models adjusted for sex, BMI, and corresponding baseline scores.

## Supplementary Table 3. Change in self-report questionnaires from week 0 to week 12 (estimated marginal means) (Per Protocol Set)

|  | | Placebo (n=41) | | | | | | Tocotrienols (n=41) | | | | | | p-value^b^ | Cohen's D |
| --- | --- | --- | --- | --- | --- | --- | --- | --- | --- | --- | --- | --- | --- | --- | --- |
|  |  | Week 0 | Week 4 | Week 8 | Week 12 | Change^b^ | p-value^a^ | Week 0 | Week 4 | Week 8 | Week 12 | Change^b^ | p-value^a^ |  |  |
| BRIEF-2: BRI (T-score) | Mean | 57.23 | - | - | 55.71 | -1.82 | 0.098 | 57.58 | - | - | 56.00 | -1.41 | 0.099 | 0.762 | 0.07 |
|  | SE | 1.50 | - | - | 1.48 | 0.94 |  | 1.70 | - | - | 1.69 | 1.00 |  |  |  |
| BRIEF-2: MI (T-score) | Mean | 61.73 | - | - | 60.52 | -1.30 | 0.167 | 58.96 | - | - | 56.58 | -2.59 | 0.006 | 0.301 | 0.24 |
|  | SE | 1.62 | - | - | 1.62 | 0.86 |  | 1.69 | - | - | 1.68 | 0.92 |  |  |  |
| BRIEF-2: GEC (T-score) | Mean | 60.34 | - | - | 59.01 | -1.45 | 0.119 | 58.93 | - | - | 56.66 | -2.34 | 0.007 | 0.456 | 0.17 |
|  | SE | 1.57 | - | - | 1.56 | 0.83 |  | 1.64 | - | - | 1.63 | 0.88 |  |  |  |
| PROMIS Sleep Disturbance (T-score) | Mean | 49.24 | 48.76 | 49.01 | 48.20 | -1.58 | 0.205 | 49.67 | 48.85 | 48.52 | 45.81 | -4.26 | < 0.001 | 0.015 | 0.58 |
|  | SE | 1.11 | 1.11 | 1.11 | 1.11 | 0.75 |  | 1.16 | 1.16 | 1.16 | 1.15 | 0.79 |  |  |  |
| PROMIS Sleep-Related Impairment (T-score) | Mean | 50.06 | 48.10 | 48.35 | 47.79 | -1.97 | 0.033 | 46.33 | 45.12 | 46.17 | 43.46 | -4.15 | 0.006 | 0.179 | 0.32 |
|  | SE | 1.37 | 1.37 | 1.37 | 1.37 | 1.10 |  | 1.41 | 1.40 | 1.41 | 1.40 | 1.18 |  |  |  |

Results (estimated means) are generated from generalised mixed-effects models adjusted for age, sex, BMI, and CTTES positive and negative scores. ^a^P-values are generated from repeated measures generalised mixed-effects models adjusted for age, sex, BMI, and CTTES positive and negative scores. ^b^P-values and change scores are generated from the change in mean scores from week 0 to week 12 using generalised mixed-effects models adjusted for age, sex, BMI, CTTES positive and negative scores, and corresponding baseline scores.

######

## Supplementary Table 4. Change in blood concentrations over time (Per Protocol Set)

|  | | Placebo | | | | Tocotrienols | | | | p-value^b^ |
| --- | --- | --- | --- | --- | --- | --- | --- | --- | --- | --- |
|  |  | Week 0 | Week 12 | Change^b^ | p-value^a^ | Week 0 | Week 12 | Change^b^ | p-value^a^ |  |
| MDA (ng/mL) | Mean | 78.44 | 69.64 | -12.29 | 0.089 | 91.27 | 75.18 | -14.28 | 0.002 | 0.75 |
|  | SE | 5.68 | 5.69 | 4.29 |  | 6 | 5.84 | 4.46 |  |  |
|  | N | 32 | 32 | 32 |  | 35 | 35 | 35 |  |  |
| Vitamin E (mg/mL) | Mean | 6.93 | 7.27 | 0.28 | 0.521 | 8.69 | 9.58 | 0.63 | 0.104 | 0.651 |
|  | SE | 1.52 | 1.53 | 0.55 |  | 1.64 | 1.67 | 0.57 |  |  |
|  | N | 32 | 32 | 32 |  | 35 | 35 | 35 |  |  |
| IL-6 (pg/mL) | Mean | 38.3 | 43.54 | 5.28 | 0.008 | 37.64 | 39.08 | 1.42 | 0.41 | 0.205 |
|  | SE | 6.41 | 6.53 | 2.11 |  | 6.36 | 6.43 | 2.2 |  |  |
|  | N | 33 | 33 | 33 |  | 36 | 36 | 36 |  |  |
| TNF-α (pg/mL) | Mean | 35.18 | 43.68 | 8.61 | < 0.001 | 29.92 | 33.16 | 3.63 | 0.079 | 0.072 |
|  | SE | 3.87 | 3.88 | 1.91 |  | 3.94 | 3.97 | 1.97 |  |  |
|  | N | 33 | 33 | 33 |  | 36 | 36 | 36 |  |  |
| IGF (ng/mL) | Mean | 161.98 | 152.89 | -5.15 | 0.179 | 147.01 | 150.8 | 1.06 | 0.585 | 0.508 |
|  | SE | 8.06 | 8.04 | 6.53 |  | 8.35 | 8.36 | 6.72 |  |  |
|  | N | 33 | 33 | 33 |  | 36 | 36 | 36 |  |  |
| BDNF (pg/mL) | Mean | 625.85 | 607.1 | -23.26 | 0.725 | 706.6 | 772.94 | 73.43 | 0.23 | 0.167 |
|  | SE | 56.75 | 56.67 | 48.09 |  | 59.39 | 59.8 | 50.31 |  |  |
|  | N | 33 | 33 | 33 |  | 36 | 36 | 36 |  |  |
| hs-CRP (mg/L) | Mean | 2.3 | 1.56 | -0.6 | 0.113 | 1.07 | 2.03 | 0.81 | 0.035 | 0.049 |
|  | SE | 0.47 | 0.45 | 0.49 |  | 0.42 | 0.46 | 0.52 |  |  |
|  | N | 35 | 35 | 35 |  | 40 | 40 | 40 |  |  |

Results (estimated means) are generated from generalised mixed-effects models adjusted for age, sex, and BMI. ^a^P-values are generated from repeated measures generalised mixed-effects models adjusted for age, sex, and BMI. ^b^P-values and change scores are generated from the change in mean scores from week 0 using generalised mixed-effects models adjusted for age, sex, BMI, and corresponding baseline scores.

######

## Supplementary Table 5. Possibly or Probably Related Adverse Events (AE) by Class and Term

| **AE Class** | **Diagnosis or symptom** | **Placebo (n=45)** | **Tocotrienols (n=46)** |
| --- | --- | --- | --- |
| Cardiovascular | **Number of participants** | **1 (2.2%)** | **0 (0.0%)** |
|  | Irregular heartbeat | 1 (2.2%) | 0 (0.0%) |
| Constitutional symptoms | **Number of participants** | **0 (0.0%)** | **1 (2.2%)** |
|  | Fatigue | 0 (0.0%) | 1 (2.2%) |
| Dermatological | **Number of participants** | **0 (0.0%)** | **1 (2.2%)** |
|  | Pruritus/ itching | 0 (0.0%) | 1 (2.2%) |
|  | Rash on leg | 0 (0.0%) | 1 (2.2%) |
| Neurological | **Number of participants** | **1 (2.2%)** | **1 (2.2%)** |
|  | Tingling in hands | 1 (2.2%) | 0 (0.0%) |
|  | Headaches | 0 (0.0%) | 1 (2.2%) |
| **Number of participants experiencing no treatment-related AEs** | | **43 (95.5%)** | **43 (93.5%)** |

*Some participants experienced more than one treatment-related AE

## Supplementary Table 6. Frequency of PGATT Responses at Week 12

|  | Placebo (n) | Tocotrienols (n) | P-value |
| --- | --- | --- | --- |
| EXCELLENT. I experienced no discomfort or adverse effects | 39 (92.9%) | 38 (86.4%) | 0.348 |
| GOOD. I experienced minimal discomfort/ side effects, but it did not interfere with my normal activities | 3 (7.1%) | 4 (9.1%) |  |
| MODERATE. I experienced moderate discomfort/ side effects, and it had some effect on my normal activities | 0 (0.0%) | 2 (4.5%) |  |
| POOR. I experienced significant discomfort/ side effects, and it significantly interfered with my normal activities | 0 (0.0%) | 0 (0.0%) |  |

* Chi-square test

## Supplementary Table 7. Change in BMI and blood pressure over time

|  |  | Placebo | | | | Tocotrienols | | | | p-value^b^ |
| --- | --- | --- | --- | --- | --- | --- | --- | --- | --- | --- |
|  |  | Week 0 | Week 12 | Change | p-value^a^ | Week 0 | Week 12 | Change | p-value^a^ |  |
| BMI | Mean | 26.18 | 26.40 | 0.11 | 0.222 | 25.68 | 25.56 | -0.04 | 0.761 | 0.728 |
|  | SE | 0.40 | 0.43 | 0.09 |  | 0.39 | 0.45 | 0.14 |  |  |
|  | N | 45 | 42 | 42 |  | 46 | 44 | 44 |  |  |
| Systolic blood pressure (mmHg) | Mean | 135.40 | 133.14 | -1.38 | 0.497 | 132.48 | 127.14 | -5.14 | 0.011 | 0.021 |
|  | SE | 3.05 | 2.86 | 2.01 |  | 2.63 | 2.26 | 1.93 |  |  |
|  | N | 45 | 42 | 42 |  | 46 | 44 | 44 |  |  |
| Diastolic blood pressure (mmHg) | Mean | 81.18 | 78.86 | -2.14 | 0.098 | 78.65 | 78.07 | -0.66 | 0.534 | 0.094 |
|  | SE | 1.67 | 1.43 | 1.26 |  | 1.01 | 1.25 | 1.05 |  |  |
|  | N | 45 | 42 | 42 |  | 46 | 44 | 44 |  |  |

^a^P-values are generated from paired-samples T-test (week 0 to week 12); ^b^P-values are generated from independent-samples T-test for change in values from week 0 to week 12.

## Supplementary Table 8. Evaluation of Clinically Meaningful/ Importance of Study Findings

| **Clinically Meaningful/ Important Study Outcome Evaluation** | | |
| --- | --- | --- |
| Population description | Adults aged 40 to 80 years with self-reported memory complaints | |
| Symptom/ disease severity | Severe  Moderately severe  Moderate  Mild  None  Not applicable  **Comments:** A non-clinical population was recruited with a likely mild to moderate severity of subjective cognitive complaints | |
| Treatment duration | 12 weeks | |
| Treatment description | Tocotrienol 50mg twice daily with food | |
| Identified symptom/ disease improvement | Improvement in non-verbal memory | |
| Safety profile | Excellent  Good  Moderate  Poor  Very poor  **Comments:** Identified an increase in hsCRP, which was not clinically significant but requires further investigation | |
| Estimated treatment cost | Less than $30 (USD) a month | |
| Patient resources required for intervention | Less than 1 minute daily | |
| Practitioner resources required | None | |
| Level of symptom/ disease resolution | Full  Moderate  Minimal  None  Not applicable | |
| Level of noticeable symptomatic improvements to participants? | Extremely noticeable improvement | Very noticeable improvement |
|  | Moderately noticeable improvement | Minimally noticeable improvement |
|  | Unlikely noticeable improvement |  |
|  | **Comments:** There are no minimally clinically noticeable thresholds for the TOMAL-2. The non-verbal memory index improved by 7.1% in the tocotrienols group and 1.4% in the placebo group, with a Cohen’s D effect size of 0.46. This memory improvement may be minimally noticeable to participants, but remains uncertain. | |
| Based on the cost-benefit analysis, can the intervention be delivered as a stand-alone intervention to achieve substantial improvement in the symptom/disease for an individual? | Definitely Yes  Probably Yes  Uncertain  Probably Not  Definitely Not  Not applicable  **Comments:** Single nutrient supplementation should not be used as a stand-alone intervention for the treatment of subjective memory complaints or cognitive decline. | |
| Based on the cost-benefit analysis, could the intervention be delivered as an adjunct intervention for symptom/disease improvement or prevention? | Definitely Yes  Probably Yes  Uncertain  Probably Not  Definitely Not  Not applicable  **Comments:** Further investigation is required. Tocotrienols supplementation presents as a promising adjunct option to diet and lifestyle interventions to support cognitive performance in generally healthy adults with subjective memory complaints. Its efficacy as an adjunct option to prevent cognitive decline requires further investigation. | |
| Other comments/ relevant information | Improvements in self-disturbance were identified, which require confirmation and further investigation in future trials | |
